# Economic Evaluation of an Area-Wide Integrated Pest Management Program to Control the Asian Tiger Mosquito in New Jersey

**DOI:** 10.1371/journal.pone.0111014

**Published:** 2014-10-22

**Authors:** Donald S. Shepard, Yara A. Halasa, Dina M. Fonseca, Ary Farajollahi, Sean P. Healy, Randy Gaugler, Kristen Bartlett-Healy, Daniel A. Strickman, Gary G. Clark

**Affiliations:** 1 The Heller School for Social Policy and Management, Brandeis University, Waltham, Massachusetts, United States of America; 2 Center for Vector Biology, Rutgers University, New Brunswick, New Jersey, United States of America; 3 Mercer County Mosquito Control, West Trenton, New Jersey, United States of America; 4 Salt Lake City Mosquito Abatement District, Salt Lake City, Utah, United States of America; 5 Monmouth County Mosquito Extermination Commission, Eatontown, New Jersey, United States of America; 6 Department of Entomology, Louisiana State University Agricultural Center, Baton Rouge, Louisiana, United States of America; 7 Agricultural Research Service, United States Department of Agriculture, Beltsville, Maryland, United States of America; 8 Bill and Melinda Gates Foundation, Seattle, Washington, United States of America; 9 Agricultural Research Service, United States Department of Agriculture, Gainesville, Florida, United States of America; Kansas State University, United States of America

## Abstract

*Aedes albopictus* is the most invasive mosquito in the world, an important disease vector, and a biting nuisance that limits outdoor activities. Area-wide integrated pest management (AW-IPM) is the recommended control strategy. We conducted an economic evaluation of the AW-IPM project in Mercer and Monmouth Counties, New Jersey with a controlled design (AW-IPM vs. control) from 2009 through 2011. The study analyzed financial documents and staff time for AW-IPM and surveyed an average of 415 randomly chosen households in AW-IPM and control areas each fall from 2008 through 2011. Hours lost from yard and porch activities were calculated as differences between actual and potential hours of these activities in an average summer week if there had been no mosquito concerns. Net estimated benefits of AW-IPM were based on cross-over and difference-in-difference analyses. Reductions in hours lost were valued based on respondents' willingness to pay for a hypothetical extra hour free of mosquitoes spent on yard or porch activities and literature on valuation of a quality adjusted life year (QALY). The incremental cost of AW-IPM per adult was $41.18 per year. Number of hours lost due to mosquitoes in AW-IPM areas between the base year (2008) and the intervention years (2009-2011) declined by 3.30 hours per summer week in AW-IPM areas compared to control areas. Survey respondents valued this improvement at $27.37 per adult per summer week. Over the 13-week summer, an average adult resident gained 42.96 hours of yard and porch time, worth $355.82. The net benefit over the summer was $314.63. With an average of 0.0027 QALYs gained per adult per year, AW-IPM was cost effective at $15,300 per QALY gained. The benefit-cost ratio from hours gained was 8.64, indicating that each $1 spent on AW-IPM gave adults additional porch and yard time worth over $8.

## Introduction

Globalization and the expansion of transcontinental shipment of tires and other goods in the 1970s led to the worldwide spread of an Asiatic mosquito species, *Aedes albopictus* (Skuse), and its establishment in new regions and countries around the globe [1]–[3]. In the United States, the first established population of *Ae. albopictus* was detected in Harris County, Texas in 1985. Since then, this species has dispersed to 36 additional southeastern and mid-Atlantic states of the United States [4]. *Aedes albopictus* is a daytime biting mosquito also known as the Asian tiger mosquito and is a nuisance [5]–[7] as well as a potential disease vector. [1], [4], [8]–[10]This species is reported to vector at least 22 arboviruses, including dengue, chikungunya, and yellow fever [1], [4], [8], [10]. The establishment of this species in the southeastern and mid-Atlantic states of the United States, combined with increasing number of travelers to arbovirus endemic countries that return infected, is a concern for both mosquito control and public health officials. This situation increases the risk of local transmission of arbovirus diseases as observed through autochthonous transmission of dengue and/or chikungunya in Hawaii, USA [11], France [12], Croatia [13], and Italy [14].

*Aedes albopictus* is currently the most invasive mosquito in the world due to its ability to thrive in both tropical and temperate climates [1]. In its native habitat in Asia *Aedes albopictus* prefers rural environments, where it is found at the edge of forests [15], [16]. In the exotic range, however, its strong ecological flexibility have resulted in rapid adaptation to urban environments where it explores a broad range of water-holding containers, especially small pockets of water in buckets and other artificial containers ubiquitous in private yards [16], [17]. *Aedes albopictus* can reach high densities and is a pestiferous biter, generating many service requests to local mosquito control programs [18]. These characteristics make area-wide integrated pest management (AW-IPM) the recommended approach for control [19], [20]. As the name indicates, AW-IPM targets hundreds or thousands of premises, obtaining economies of scale and reducing re-infestation from untreated areas. Until recently such widespread mosquito control has been unusual in urban areas except during disease outbreaks where adulticides are used area-wide. In contrast, AW-IPM is preemptive and includes source reduction, directly and through education campaigns, application of larvicides and, if needed, adulticides [20]. AW-IPM has been successful in eradicating the early introduction of *Ae. albopictus* from New Zealand [18] as well as in reducing the population of fire ants, fruit flies, stored-grain insects, leafy spurge, corn rootworm, and codling moth [21]–[27]. The consolidated industry net benefits associated with the Hawaii fruit fly AW-IPM program alone were projected for the year 2015 at US$5.8 billion [28].

Economic evaluation is important to understand and quantify the benefits associated with a publicly funded program, and to inform the decision making process addressing the allocation of scarce public resources among projects that contribute most to the welfare of society [29]. While the potential public health threat of *Ae. albopictus* is alarming, the nuisance associated with this day biter and its impact on residents daily activities is rarely studied [7], [30]. As part of the AW-IPM program for the control of *Ae. albopictus* in Mercer and Monmouth Counties in New Jersey [20], [31], this paper presents a benefit-cost analysis of this project's activities from 2009 through 2011 to mitigate the mosquito's nuisance. It also assesses the cost-effectiveness of these activities in improving residents' utility in study areas.

## Methods

### Impact of project activities

#### Study design

We used two economic evaluation techniques (i.e., benefit-cost and cost-effectiveness) to quantify the economic impact of the AW-IPM from a societal perspective. For the benefit-cost analysis of this project, we computed the economic cost of project activities, derived the household benefit from the AW-IPM by computing the reduction in the number of yard and porch hours lost in a typical summer week due to mosquitoes, and quantified the value associated with one additional mosquito-free-hour engaged in yard and porch activities. To measure the cost-effectiveness of this project, we estimated the improvement in residents' mosquito-reduction utility from the AW-IPM, the quality adjusted life years (QALY) gained due to AW-IPM, and the benefit-cost ratios of the AW-IPM.

The study used a three-arm experimental design with three matched areas in each county. These were initially assigned to full intervention (AW-IPM), education intervention only, and control arms [31]. Due to challenges in gaining permission to conduct classroom activities, the education arm was discontinued, and after 2009 the two sites were repurposed for testing new products and other select interventions. Therefore, for this analysis we focus only on the AW-IPM intervention and control sites.

#### Household survey design

To quantify the benefit associated with the project activities, we conducted four annual rounds of mailed household surveys, starting with the pre-intervention year of 2008 and the three intervention years of 2009 through 2011. These surveys focused on the impact of mosquitoes on the number of hours households spent in yard and porch activities. We computed the hours lost due to mosquitoes as the difference between the actual hours respondents spent engaged in yard and porch activities in a typical summer week compared to potential hours they would have spent if there had been no concern about mosquitoes.

#### Sample frame

The sample frame of households was created from public records gathered from two sources. In Mercer County we used lists of all parcels provided by the Mercer County Planning Division. In Monmouth County, we began with a digital database of the tax assessor maintained by the Monmouth County Office of Geographic Information Systems. The Monmouth County Mosquito Extermination Commission prepared a subset limited to parcels within the study areas. Researchers at Brandeis University edited it further to include only residential addresses. Merging addresses from both sources created a sample frame of 3,986 households. We corrected the mailing addresses using US postal services Zip Code lookup website, Google Maps and Google Earth [6], [31].

#### Instrument

The self-administered mail surveys consisted of questions related to the respondent and a selected child, if present. The selected child is the only child or the eldest child in elementary school in the household. Survey questions assessed: (1) the time a respondent and the selected child, if present, spent in different yard activities; (2) the time the respondent and the selected child, if present, would have spent in various yard activities if they had no mosquito concerns; (3) awareness of *Ae. albopictus*; (4) engagement in recommended mosquito control prevention and control activities; (5) expenditure on mosquito control; (6) household experience with mosquitoes during an average summer week; and (7) household demographics.

We focused on five yard and porch activities: (1) eating and cooking in yard, (2) gardening or mowing lawn, (3) maintaining house or car, (4) playing, (5) relaxing. We added an “others” category, where we asked respondents to specify the activity. Instruments were offered in both English and Spanish. After the pre-intervention year (2008), a few questions were added to capture the impact of the educational activities; the final questionnaire can be found in Survey S1.

#### Survey implementation

Four rounds of household surveys were conducted starting in 2008. For the pre-intervention year, using systematic random sampling, we asked 900 households to respond to a mailed survey. For the intervention years, we employed a mixed sampling method: we invited households who responded to the previous surveys to participate in the subsequent surveys, and randomly selected new households to participate in the new rounds. The sample sizes for the intervention years were 1,000 for 2009, 1,167 for 2010, and 1,333 for 2011.

The surveys were mailed to the selected households the first week of October of each study year, with a cover letter explaining the objectives, importance, and possible impact of the project. A five-dollar bill was enclosed with the letter in appreciation for and recognition of the value of respondents' time (pre-incentive). In November we sent another letter and a copy of the survey for non-respondents encouraging them to complete and return the survey. Graduate students at Brandeis University coded the survey responses and entered the data into Excel spreadsheets (Microsoft Corporation, Redmond, WA). Twenty percent of the surveys were reentered to validate data entry. Data were then transferred to STATA (College Station, TX) [32] for analysis.

#### Data cleaning

We cleaned the data to identify and check outliers and inconsistent information. To clean the “hours responder/selected child spent engaged in various yard and porch activities” and “hours responder/selected child would have spent in various yard and porch activities,” we followed three steps: (1) all hours related to activities in “others” category were integrated into the five yard and porch activities when applicable, otherwise these hours were excluded since most responses referred to outdoor activities not related to yard or porch, such as going to the beach or walking the dog, (2) all missing values were coded as zero since respondents generally skipped questions that did not apply to them, and (3) outliers in hours spent or would have been spent were winsorized to the activity's 95 percentile (on average 1.85 observations per activity). For expenditures on capital items, such as installing window screens, we amortized the expense according to its expected lifetime. For fixing leaky pipes and repairing or installing screens on doors and windows to keep mosquitoes out of the home, we used 10 years, and for commercially available mosquito traps we used 3 years based on the Internal Revenue Manual [33].

#### Impact analysis

In 2011, new control strategies were introduced in Monmouth County. To make sure effects from the previous years' interventions (especially on early season activities) did not linger and affect the results, and therefore to provide the most conservative estimate of the new intervention strategies' success, the prior years' intervention and control sites were switched. This decision was experimentally conservative since any residual effects from previous years' control efforts would now be reflected in lower numbers of mosquitoes in the control sites.

To address the changes in the study sites, we used cross-over and difference-in-differences analyses to estimate the impact of the AW-IPM project's activities on the yard and porch hours lost due to mosquitoes. We pooled the four surveys in one data set to increase statistical power. We tested for any differences in the baseline populations between the pre- and post-intervention years using Chow tests [34]. To address clustering due to some residents participating in more than one survey, we used a pooled ordinary least squares (OLS) regression with cluster-robust standard errors. Our model was:
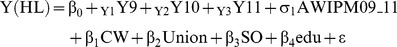



where Y(HL) refers to the change in hours lost engaged in each of the five specified yard and porch activities. The variables Y9, Y10, and Y11 are dummy variables equal to one if the observations came from the intervention years of 2009, 2010, and 2011, respectively, and zero if they came from the pre-intervention year 2008 or another year. The variable AWIPM09_11 is a dummy variable equal to one if the observations came from intervention sites for each of the intervention years and zero if it came from control sites. The variables CW, Union, and SO are dummy variables equal to one if the observation came from the Monmouth intervention site (Cliffwood Beach), Monmouth control site (Union Beach), and Mercer intervention site (South Olden), respectively, according to their base year assignment, and zero if the observation came from Mercer control site (Cummings). The variable edu is a dummy variable where one refers to the attainment of a bachelor's degree or higher and zero otherwise.

The year 2010 was unusual because that summer was the warmest and driest on record in southern New Jersey; the monthly average rainfall was 40% below the norm (2.49 inches in 2010 compared to the norm of 4.16 inches) [35]. As a result, mosquito populations in Mercer County's urban sites were reduced and residents were able to enjoy more outdoor activities. While the same level of mosquito reduction was not evident in the more humid coastal sites in Monmouth County, our results showed a reduction in the number of hours lost due to mosquitoes in these sites. Additionally, in 2010 the AW-IPM project implemented a record number of novel control and surveillance interventions, mostly on a one-time basis (Fonseca et al unpublished data, 2014). Their impact, if any, on mosquitoes during this unusual year would not be representative of other years [20]. For these reasons, we performed a sensitivity analysis that excluded the year 2010 data from our regression models, estimating the equation below:
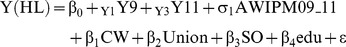



Other results are reported as un-weighted means, standard deviations, standard errors of the means, confidence intervals (CI) at 95% level for continuous variables, and frequencies for categorical variables. We performed *t*-tests and Chi-square tests, with alpha levels of significance at 0.05, for hypothesis testing. Additionally, to address a possible gender bias due to our sampling strategy which focused on women, we ran the models using a post-survey household weight including respondents' age and gender [36].

### Ethics statement

The investigators sent a cover letter to selected residents with the survey instrument stating the study objectives, potential benefits, and contacts for the investigators and Brandeis University Institutional Review Board (IRB) in case of any concerns. Subjects were informed their response was voluntary and were compensated five dollars in appreciation for their time. Participants indicated their consent by submitting a completed survey. We removed from the sample frame the names of residents who contacted us to state their lack of interest in the survey. The IRB at Brandeis University reviewed and approved the research protocol (IRB number: 09012).

### Economic cost of vector control

We ascertained the facility-based cost of operational surveillance and vector control activities for Mercer and Monmouth Counties as well as Rutgers University's education component for the duration of the project, starting with the pre-intervention year of 2008. We developed a costing questionnaire to capture the cost of all resources, including donated items, used for *Ae. albopictus* operational surveillance and control in the selected project sites. We reviewed financial records to determine the cost of personnel, materials, and amortized capital items. For donated and some capital items we used the market value to estimate their cost. We amortized capital items according to their useful lives based on the Internal Revenue Manual [33] with a discount rate of 3%. Interviews with county officials assisted in distributing resources (1) between *Ae. albopictus* surveillance and control measures for other mosquitoes and (2) by function, differentiating *Ae. albopictus* AW-IPM program resources from routine county *Ae. albopictus* control activities.

### Benefit-cost and cost-effectiveness analyses

Benefit-cost analysis is one form of economic evaluation where both the costs and benefits of a project are evaluated in monetary terms [37]. If the benefit-cost ratio associated with the project exceeds 1, then it will be considered beneficial and recommended. To compute the benefit associated with AW-IPM project, we measured the effectiveness of the AW-IPM project in reducing the number of hours lost per yard and porch activity due to mosquitoes. To estimate the monetary value of yard and porch hours gained due to the AW-IPM project, we used the monetary values associated with the estimated gain generated from contingency valuations in face-to-face interviews conducted in the study AW-IPM and control areas in 2010 [38]. To estimate the benefit-cost value associated with the AW-IPM for all years we used a pooled OLS regression with cluster-robust standard errors model:



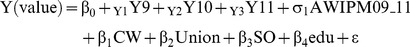
where the “value” variable is the sum of hours gained per observation for each yard or porch activity multiplied by the hourly value attributed to that activity. As a sensitivity analysis, we ran another model excluding the data for 2010: 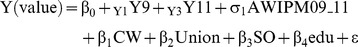



#### Cost- effectiveness analysis

In this form of economic evaluation both the costs and the quality of life of health or health-related interventions are assessed [37]. From the cost analysis we computed the incremental cost of the program and compared it to the incremental improvement in quality of life attributed to the AW-IPM project. The improvement is measured by the gain in health outcome using QALYs, a single metric that captures two important dimensions of intervention outcomes: the degree of improvement and the time interval over which the improvement occurs. It enables us to compare the efficiency of health or health-related interventions using the same units. The QALY assumes a year lived in perfect health is worth 1 QALY (1 year of life × 1 utility value), and that a year lived in a state of less than perfect health is valued less than 1 [37].

To measure the QALY gains from the AW-IPM during the summer of 2010, we derived the incremental gain in the mosquito-reduction utility [38] by subtracting the mosquito-reduction utility for individuals living in the AW-IPM areas from the utility in the control areas. For this analysis we assumed that residents were exposed to mosquitos during the summer, which we defined as 91 calendar days (from June to August), or 25% of the year. To compute the incremental annual QALYs, we multiplied 25% times the utility gain during the summer [38] To calculate the cost-effectiveness ratio, we divided the incremental cost by the incremental benefits associated with the AW-IPM.

## Results

### Household surveys

#### Sample size

Of the 4,400 questionnaires sent out from 2008 through 2011, 1,659 questionnaires (38%) were returned from 977 households. The study design and response rates yielded a sample where 60% of households responding for one year of the four annual surveys, 20% for two years, 12% for three, and 9% responded to all four years. The response rates for all sampled addresses were 34%, 39%, 35%, and 41%, and the rates after excluding undelivered letters were 42%, 43%, 39%, and 48% for the years 2008, 2009, 2010, and 2011, respectively.

#### Household characteristics

The majority (60%) of the 977 respondents resided in Monmouth County, had less than a bachelor's degree (81%), were women (76%), were in the labor force (59%), and were between the ages of 25–54 years (53%). On average (± standard deviation [SD]) their household size was 2.8 (±1.5) persons, and 35% of respondents had at least one child under 18 years of age. Table 1 compares respondents' characteristics with those of households in the study areas. We found no statistically significant differences in having a child at home, household size, or employment status. However, we found statistically significant differences between counties in the respondents' gender, age, and education.

**Table 1:** Household characteristics of respondents compared to study sites†.

Variable	Sample population	Study sites	Sig.
Number of households in each county (N = 977)			
Mercer	40%	33%	**
Monmouth	60%	67%	
Child at home^+^ (N = 977)			
Household with one or more people under 18 years	35%	35%	NS
Respondent's gender (N = 948)			
Female	76%	51%	***
Respondent's age (N = 964)			
35–44	27%	19%	***
45–54	28%	19%	
55–64	24%	21%	
65–74	12%	23%	
75 up	9%	18%	
Respondent level of education# (N = 933)			
Less than 9th grade	2%	7%	***
9–12 grade	8%	9%	
High school graduate	37%	34%	
Some college no degree	26%	19%	
Associates degree	8%	8%	
Bachelor's degree	12%	16%	
Graduate or professional	7%	8%	
Average household size (N = 977)	2.78	2.69	NS
Respondent employment status (N = 944)			
In the labor force	59%	60%	NS
Unemployed looking for a job	5%	7%	
Not in labor force	36%	33%	

† For discrete categories, entries denote that category as a % of the total (column percentages); for continuous variables, entries are the means.

* P<0.05; **P<0.01; ***P<0.001; NS =  Not statistically significant.

+ child under 18.

# Population 25 years and over.

#### Benefits of the project

The average (±SEM) number of hours an adult resident spent in yard and porch activities during a typical summer week in baseline year (2008) did not differ significantly between AW-IPM (9.09±1.01) and control areas (6.50±0.83), suggesting the areas were comparable. The average hours lost in 2009 through 2011 were 16.85±0.88 in the AW-IPM areas compared to 18.71±0.88 in the control areas. Table 2 presents the average hours lost by activity and year. Overall, the AW-IPM program recouped 4.45 previously lost hours (standard error is 1.80, p<0.01).

**Table 2:** Difference in differences in key outcomes in a typical summer week for adult residents.

	Hours lost						Value lost due to mosq. ($)	Expenditure on mosquito control ($)
	Eat-ing	Gar-dening	Main-taining	Play-ing	Relax-ing	Total hours		
Year 2008								
AW-IPM								
Mean	2.78	1.45	0.65	1.06	3.62	9.09	91.02	54.37
SEM	0.36	0.22	0.14	0.19	0.45	1.01	9.55	7.45
Control								
Mean	2.03	1.11	0.45	0.89	2.54	6.50	66.43	48.04
SEM	0.27	0.19	0.11	0.18	0.33	0.83	7.85	9.12
Average 2009–2011								
AW-IPM								
Mean	4.26	2.48	1.85	3.12	5.60	16.85	158.49	43.15
SEM	0.25	0.18	0.13	0.18	0.35	0.88	8.11	4.89
Control								
Mean	4.60	3.06	2.30	3.35	5.77	18.71	172.91	31.16
SEM	0.25	0.19	0.14	0.17	0.33	0.88	8.04	1.81
Difference in differences+								
Mean	−1.09*	−0.91**	−0.65**	−0.41NS	−1.25*	−4.45**	−39.01*	5.66NS
SEM	0.57	0.39	0.26	0.36	0.74	1.80	16.83	12.88

Notes: AW-IPM denotes area-wide integrated pest management; SEM denotes standard error of the mean; NS denotes not statistically significant; mosq denotes mosquito; * p<0.05, ** p<0.01, + Negative values denotes a favorable outcome.

We compared the hours lost due to mosquitoes for the selected child under the age of 18 (N = 561). On average (±SD) in 2008, before the AW-IPM intervention, a child lost 17.63±20.58 hours per week due to mosquitoes in AW-IPM areas compared to 10.21±13.46 hours in control areas (t(117) = −2.36, p = 0.020). This significant difference indicates the importance of controlling for baseline differences between areas for children. Our cross-over and difference-in-differences analyses suggest that AW-IPM activities had no impact on the total hours a selected child lost due to mosquitoes.

The average expenditure on personal and household mosquito control activities in 2008 was $54.3±88.20 in the AW-IPM areas, and $48.04±118.90 in the control areas. We found no statistically significant differences in expenditures between the study areas in 2008, indicating that the areas were well matched. The cross-over and difference-in-differences analyses showed no AW-IPM project overall impact on household expenditure on mosquito control activities.

### Economic cost of vector control

Table 3 presents the annual cost of *Ae. albopictus* control activities in AW-IPM and control areas. The average cost of the AW-IPM activities for the years 2009 through 2011 was $35.90 per capita and $44.34 per adult. The average incremental cost (the cost of controlling *Ae. albopictus* in the intervention areas minus the cost of controlling *Ae. albopictus* in the control areas through routine county control activities) was $33.34 per capita and $41.18 per adult (see Table 3).

**Table 3:** Cost of controlling *Aedes albopictus* in Mercer and Monmouth Counties in intervention and control areas, 2008–2011.

Intervention areas	2008	2009	2010	2011	Average (2009–2011)
***AW-IPM***					
Mercer	$9,763	$179,846	$186,054	$107,266	$157,722
Monmouth †	$1,541	$106,882	$129,042	$76,385	$104,103
Rutgers (education)	$0	$39,655	$50,312	$9,780	$33,249
Total	$11,304	$326,384	$365,409	$193,431	$295,075
Population (all ages)^Υ^	8,220	8,220	8,220	8,220	8,220
Adult population (15+)	6,655	6,655	6,655	6,655	6,655
Cost per capita	$1.38	$39.71	$44.45	$23.53	$35.90
*Cost per adult (15+)*	*$1.70*	*$49.04*	*$54.91*	*$29.07*	*$44.34*
***Control***					
Mercer	$9,763	$8,475	$3,026	$62,071	$24,524
Monmouth a	$1,541	$1,568	$1,535	$13,894	$5,665
Rutgers (education)	$0	$0	$0	$9,780	$3,260
Total	$11,304	$10,043	$4,561	$85,745	$33,449
Population (all ages)^&^	13,083	13,083	13,083	13,083	13,083
Adult population (15+)	10,592	10,592	10,592	10,592	10,592
Cost per capita	$0.86	$0.77	$0.35	$6.55	$2.56
*Cost per adult (15+)*	*$1.07*	*$0.95*	*$0.43*	*$8.10*	*$3.16*
***Incremental cost per adult (15+)***	***$0.63***	***$48.10***	***$54.48***	***$20.97***	***$41.18***

† In 2011 the former control area was converted to an AW-IPM area; ^Υ^ denotes area-wide integrated pest management (AW-IPM) population in both Mercer and Monmouth counties.

a In 2011 the former AW-IPM area was converted to a control area; ^&^ denotes control areas population in both Mercer and Monmouth counties.

During the intervention years (2009–2011), the AW-IPM project spent on average 20% ($43,448) of total costs on adulticiding, 28% ($60,407) on larviciding, 16% ($35,551) on source reduction, 25% ($53,587) on surveillance, and 11% ($24,455) on education. In comparison, the counties' routine *Ae. albopictus* control activities were allocated as follows on average: 23% ($27,878) was spent on adulticiding, 28% ($34,110) on larviciding, 18% ($22,629) on source reduction, 17% ($20,888) on surveillance, and 14% ($17,739) on community education campaigns. The majority of the costs supported personnel (representing 68% of AW-IPM costs and 63% of routine costs), followed by recurrent costs (23% of AW-IPM, 26% of routine activities), and amortized capital costs (9% of AW-IPM, 11% of routine activities).

### Benefit-cost and cost-effectiveness analyses

#### Analysis based on hours gained

**Figure 1:**
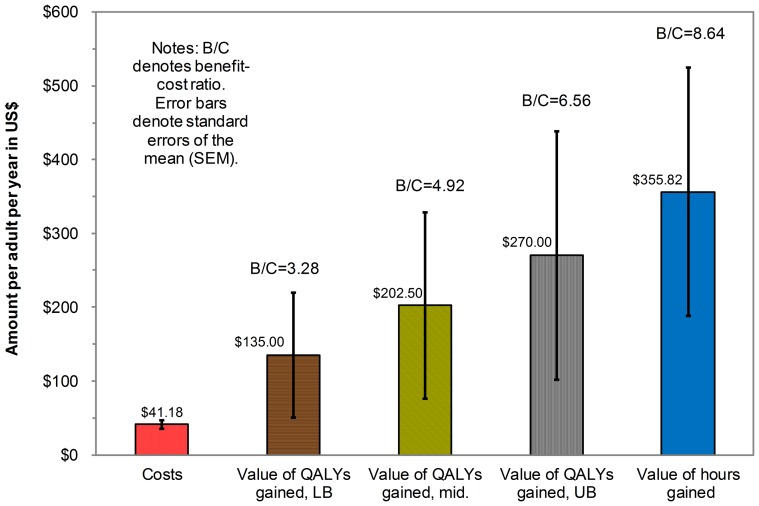
Costs and net benefits per adult resident of the AW-IPM project based on the value of the hours and QALYs gained. Note: LB denotes lower bound, mid. denotes midpoint; UB denotes upper bound.

From the Chow test we conclude that there were no significant differences between the sampled populations during the study period in respondents' characteristics. As presented in Table 4, the AW-IPM project reduced the average (±standard error of the mean [SEM]) total hours lost per summer week engaged in a yard or porch activities due to mosquitoes in the AW-IPM areas by 3.30±1.41 hours per week compared to control areas; this difference was statistically significant (p = 0.02). These hours gained (i.e., the reduction in hours lost) translated to residents' statistically significant (p = 0.04) perceived benefit of $27.37±12.95 per week, with a 95% CI of $1.97 to $52.78. This improvement translated to 42.96 hours gained over the 13-week summer (3.30 times 13) and a monetary valuation per adult resident of $355.82 per year (CI: $25.61 to $686.14). The incremental cost per adult of AW-IPM was $41.18 per year and the benefit-cost ratio was 8.64 (CI: 0.62 to16.66), as shown in Figure 1.

**Table 4:** Pooled ordinary least squares (OLS) regression with cluster-robust standard errors of the hours lost per week enjoying yard and porch activities due to mosquitoes, and the value lost due to mosquitoes, 2008–2011 (base case).

Indep. variables	Eating		Gar-dening		Main-taining		Play-ing		Relax-ing		Total hours		Value lost due to mosq. ($)		Reference category
Study years															
Year 2009	3.22	***	2.85	***	2.59	***	3.66	***	4.86	***	17.17	***	151.16	***	Year 2008
	(6.95)		(8.05)		(10.50)		(11.55)		(7.50)		(10.63)		(10.13)		
Year 2010	0.07		0.22		0.41	**	0.02		−0.03		0.76		4.94		
	(0.22)		(1.06)		(2.82)		(0.10)		(0.07)		(0.78)		(0.53)		
Year 2011	3.49	***	2.68	***	2.42	***	3.40	***	4.01	***	16.13	***	140.60	***	
	(8.15)		(8.56)		(11.22)		(12.14)		(7.13)		(11.34)		(10.72)		
Assigned intervention areas	−0.42		−0.94	**	−0.68	**	−0.35		−0.82		−3.30	*	−27.37	*	Assigned control areas
	(1.03)		(3.06)		(3.11)		(1.25)		(1.44)		(2.35)		(2.11)		
Study areas															
South Olden (AW-IPM_baseline)	0.17		0.65		0.50	+	0.00		0.95		2.28		20.27		Cummings (Mercer control area)
	(0.30)		(1.55)		(1.82)		(0.01)		(1.26)		(1.21)		(1.16)		
Cliffwood Beach (AW-IPM_baseline)	−0.03		0.50		0.21		0.36		0.88		1.96		16.99		
	(0.06)		(1.51)		(0.89)		(1.16)		(1.44)		(1.26)		(1.19)		
Union Beach (control_baseline)	−0.40		0.21		0.23		0.07		0.25		0.40		2.05		
	(0.93)		(0.71)		(1.05)		(0.23)		(0.45)		(0.29)		(0.16)		
Attainment of a B.A. degree or higher	−0.96	**	−0.62	*	−0.01		−0.64	*	−0.98	*	−3.18	**	−29.99	**	Respondents with less than BA degree
	(2.82)		(2.40)		(0.04)		(2.53)		(2.08)		(2.74)		(2.84)		
Constant	2.49	***	1.00	***	0.31	*	0.94	***	2.64	***	6.91	***	70.99	***	
	(7.00)		(4.21)		(1.98)		(4.12)		(5.99)		(6.30)		(6.89)		
Effectiveness of AW-IPM	0.42		0.94		0.68		0.35		0.82		3.30		27.37		
Hours lost in 2008	2.78		1.45		0.65		1.06		3.62		9.09		77.54		
% gained due to AW-IPM	15.0%		64.6%		104.5%		33.1%		22.7%		36.4%		35.3%		
Observations	1581		1581		1581		1581		1581		1581		1581		
R-square	7.67%		8.35%		11.54%		16.66%		7.19%		13.88%		12.95%		

Note: t-value in parentheses (ignoring sign). AW-IPM denotes area-wide integrated pest management. Indep. denotes independent. Mosq. denotes mosquitoes.

+ p<.10, * p<0.05, ** p<0.01, *** p<0.001.

As expected, the share of respondents who reported that the presence of mosquitoes prevented them from enjoying their porch and yard activities somewhat or very much varied by year. The percentages were 65.05% in 2008, 58.4% in 2009, 46.47% in 2010, and 60.89% in 2011. These differences among years were highly significant (*X^2^* (9)  = 58.62, p<0.001), confirming the value of the sensitivity analysis excluding year 2010. As shown in Table 5, under that sensitivity analysis, the AW-IPM reduced the average (±SEM) hours lost per summer week engaged in a yard or porch activities due to mosquitoes in the AW-IPM areas by 5.40±1.79 hours per week compared to control areas; this difference was statistically significant (p = 0.003). The hours gained were associated with residents' perceived benefit of $45.85±16.50 (p = 0.006) (CI: $13.45 to $78.24). This improvement translated to 70.17 hours gained over the 13-week summer and a monetary valuation per adult resident of $595.99 per year (CI: $174.89 to $1,017.09). The net benefit from the AW-IPM is $554.81 (CI: $133.71 to $975.91).

**Table 5:** Pooled ordinary least squares (OLS) regression with cluster-robust standard errors of the hours lost per week enjoying yard and porch activities due to mosquitoes, and the value lost due to mosquitoes, for the years 2008, 2009, and 2011 (sensitivity analysis).

Indep. variables	Eating		Gar-dening		Main-taining		Play-ing		Relax-ing		Total hours		Value lost due to mosq. ($)		Reference category
Study years															
Year 2009	3.41	***	3.04	***	2.75	***	3.80	***	5.11	***	18.12	***	159.41	***	Year 2008
	(6.93)		(7.95)		(10.26)		(11.25)		(7.43)		(10.43)		(9.96)		
Year 2011	3.67	***	2.86	***	2.57	***	3.54	***	4.24	***	17.02	***	148.30	***	
	(8.02)		(8.47)		(10.98)		(11.80)		(7.01)		(11.02)		(10.41)		
Assigned intervention areas	−0.83		−1.35	**	−1.03	***	−0.67	+	−1.41	+	−5.40	**	−45.85	**	Assigned control areas
	(1.63)		(3.40)		(3.66)		(1.83)		(1.94)		(3.01)		(2.78)		
Study areas															
South Olden (AW-IPM_baseline)	0.67		0.85	+	0.62	+	0.17		1.25		3.45		32.24		Cummings (Mercer control area)
	(1.01)		(1.72)		(1.86)		(0.38)		(1.40)		(1.54)		(1.57)		
Cliffwood Beach (AW-IPM_baseline)	−0.07		0.43		0.14		0.31		0.95		1.74		16.09		
	(0.12)		(1.13)		(0.52)		(0.88)		(1.37)		(0.97)		(0.97)		
Union Beach (control_baseline)	−0.02		0.52		0.48		0.31		0.87		2.11		18.45		
	(0.04)		−1.26		(1.61)		(0.81)		(1.19)		(1.11)		(1.06)		
Attainment of a bachelor degree or higher	−1.22	**	−0.62	+	0.07		−0.87	**	−1.08	+	−3.80	*	−35.02	**	Respondents with less than B.A.
	(2.89)		(1.86)		(0.27)		(2.72)		(1.80)		(2.56)		(2.60)		
Constant	2.36	***	0.92	**	0.24		0.91	**	2.44	***	6.47	***	66.12	***	
	(5.92)		(3.23)		(1.22)		(3.42)		(4.84)		(5.00)		(5.51)		
Effectiveness of AW-IPM	0.83		1.35		1.03		0.67		1.41		5.40		45.85		
Hours lost in 2008	2.78		1.45		0.65		1.06		3.62		9.09		77.54		
% gained due to AW-IPM	29.8%		93.3%		158.8%		63.6%		38.9%		59.4%		59.1%		
Observations	1185		1185		1185		1185		1185		1185		1185		
R-square	5.34%		5.64%		8.61%		11.07%		4.64%		9.48%		8.77%		

Notes: t-value in parentheses (ignoring sign). AW-IPM denotes area-wide integrated pest management. Indep. denotes independent; Mosq. Denotes 'mosquitoes. Negative values denote favorable outcomes.

+ p<.10, * p<0.05, ** p<0.01, *** p<0.001.

#### Analysis based on improvement in quality of life

During the three summer months, the mosquito-abundance-utility score of individuals living in the AW-IPM areas was (mean±SEM) 0.8753±0.0033, compared to 0.8645±0.0059 in control areas (t(80) = 1.68, one-sided p = 0.048). The annual difference is 0.0027 QALYs, i.e., (0.8753–0.8645) × (3/12). This impact translated into a cost-effectiveness ratio of $15,300 per QALY gained per adult, (i.e. $41.18/0.0027) with a CI of $10,500 to $28,200. Using the decision rule based on the relatively low value ($50,000) of a QALY [39], we estimated the economic value of the improvement in utility from AW-IPM each year as 0.0027 × $50,000 or $135. Using the higher value of $100,000 per QALY [40], [41] and the midpoint value of $75,000 per QALY show economic benefits of AW-IPM of $270.00 and $202.50, respectively. The three-year corresponding benefit-cost ratios are 3.28, 6.56, and 4.92 respectively.

## Discussion

To our knowledge, this study is the first to estimate the benefits and effectiveness of an AW-IPM intervention from residents' perspective. Our analyses for the intervention years show a favorable impact of AW-IPM. The AW-IPM reduced the average number of hours lost due to mosquitoes between 2009 and 2011 by 3.30 hours per summer week; indicating a 36.4% reduction in hours lost due to mosquitoes in the intervention areas compared to control areas. While the impact was statistically significant, we noticed substantial variation in the hours gained: coefficient of variation of 16.95 (CI: 0.55−6.06). This variation is due to differences in the hours lost among the study years. During the years of 2009 and 2011, when the summer weeks in southern New Jersey were far wetter than the 1981−2010 historical average, the hours lost due to mosquitoes were significantly higher. In contrast, the hours lost in 2010, the driest summer on record [35], were negligible and insignificant. This finding is consistent with nuisance levels reported by respondents; 60.89% reported that mosquitoes prevented them from enjoying their outdoor activities in 2011, compared to 46.47% in 2010. When we modeled the impact of AW-IPM excluding the year 2010, the coefficient of variation decreased to 11.44, and the overall hours gained per summer week increased to 5.40 in the intervention areas compared to control areas. This finding suggests that AW-IPM might be more effective if implemented during abnormally wet years.

The hours of yard and porch activities gained due to AW-IPM were valued at $27.37 (CI: $1.97−$52.78) per adult-resident per summer week, or $355.82 (CI: $25.61−$686.10) per adult-resident over the 13-week summer for improvement in the quality and enjoyment of their yard and porch activities. Excluding the year 2010, the result shows a 59.4% reduction in the number of hours lost compared to the base year 2008, due to the AW-IPM activities. This benefit is valued at $595.99 (CI: $174.89 − $1,017.09) per adult-resident over the 13-week summer.

Our previous study [38] showed that residents were willing to pay on average (±SEM) $8.53±$1.13 for an additional hour spent engaged in yard and porch activities without mosquitoes. Hypothetically, if the project succeeded in eliminating all mosquitoes in the AW-IPM, then – assuming there is a constant marginal utility for each additional hour and no budget constraint – adult residents would have been willing to pay $144 per summer week or $1,868 over the 13-weeks summer to enjoy their yard optimally. However, *Ae. albopictus* is so invasive that once the species is established in an area, its eradication is virtually impossible; but control efforts can reduce the species' nuisance to an acceptable level [20]. Our results indicate that adult residents in the AW-IPM areas gained nearly 36.4% of the value lost in 2008 engaged in yard and porch activities due to the AW-IPM intervention during the AW-IPM intervention years, and a 59.1% during the Years 2009 and 2011.

In a prior study, we quantified the impacted of mosquitoes, including the urban mosquito *Ae. albopictus*, on residents' quality of life using a mosquito-abundance-utility scale [38]. That study demonstrated an improvement in the mosquito-abundance-utility scale, which translates to an improvement of 0.0108 in utility scale and 0.0027 in QALY gained per adult. The cost-effectiveness analysis yields a cost-effectiveness ratio of $15,300 per QALY gained per adult. This result is considered very cost effective using the cost-effectiveness threshold of per capita GDP [37] (i.e., $49.854 for the United States in 2011) [42].

The costing and evaluating of the project's benefits and effectiveness were measured while the AW-IPM experimental phases were underway. This allowed us to differentiate the personnel effort and resources allocated to control *Ae. albopictus* through routine county and AW-IPM activities from those related to research activities. These interventions served all residents in the selected AW-IPM areas, but we found benefits only for adult residents. In 2011, the project's educational component, which included a countywide school educational campaign, door-to-door education activities, and public service announcements, was extended to control areas. The expansion of the educational component contributed to an increase in the overall cost per adult in the control areas in 2011 (from an average of $0.69 in 2009−2010 to $8.10 in 2011). Additionally, as the project matured and control protocols were further tested and developed, the team was able to reach a technically effective but less expensive strategy focusing on the early spring months to delay and limit adult populations, and using truck-mounted area-wide applications of larvicides and adulticides combined with active education strategies instead of costly door-to-door source reduction campaigns. These developments reduced the cost of the third intervention year (2011) compared to the first two intervention years. These two factors resulted in a reduction in the AW-IPM per adult incremental cost from an average of $51.29 in 2009−2010 to $20.97 in 2011. Focusing specifically on the mature version of the program (year 2011), the incremental cost was $20.97. Estimating the net benefit under this lower cost gives a net benefit of $334.85 (CI: $4.64 to $665.17) over the 13-week summer with a benefit cost ratio of 16.97 (CI: 1.22 to 32.72).

Our study has several strengths. First, we obtained consistently favorable results under three different measures of AW-IPM effectiveness. These measures covered quality of life, yard and porch hours, and added monetary value gained from increased yard and porch activities. All approaches showed favorable average cost-benefit ratios (i.e., above 1.00), ranging from 3.28 to 8.64. Second, in the two years when the rainfall exceeded the normal range, the program was both significantly effective and cost-effective.

Some limitations of our study must be acknowledged. First, our sampling strategy caused men to be under-represented in our surveys. However, our analysis was able to correct for this imbalance both by weighting and incorporating covariates in regression and confirmed that the imbalance did not affect our results. Second, our estimates of the incremental cost of the AW-IPM program may be overstated because they include a combination of experimental approaches during 2009−2010. If we relied exclusively on the year 2011 for cost analysis, the cost per adult resident became $20.97, the project impact $595.90, and the benefit-cost ratio 28.42. Third, we used self-report rather than advanced technologies, such as GPS and motion detectors, to estimate the precise time individual spent, or potential time lost engaged in yard and porch activities due to mosquitoes. Such advanced technologies were not feasible in this study due to their requirements for implementing a prospective design, obtaining permission from households for this intrusion to their privacy, and ensuring participants' compliance in wearing and maintaining the chosen device. While future prospective studies could consider such technologies, the expense and challenges in securing high participation and ensuring participants' compliance in wearing and maintaining the chosen device would likely be substantial.

In conclusion, the types of benefits observed on outdoor activities and residents' quality of life are consistent with projections from a recent conceptual model [30]. Our results showed significant improvements and favorable benefit-cost ratios associated with the AW-IPM project, based on assessment of hours gained enjoying yard and porch activities, and improvement in quality of life.

## Supporting Information

Survey S1 **NJ Mosquito Control Questionnaire.**
(PDF)
