# Supplementary material for: Economic Evaluation of an Area-Wide Integrated Pest Management Program to Control the Asian Tiger Mosquito in New Jersey
Source: PLoS One. 2014 Oct 22;9(10):e111014. doi: 10.1371/journal.pone.0111014 (PMC4206470; doi:10.1371/journal.pone.0111014)
Supplement: Survey S1 — NJ Mosquito Control Questionnaire. (PDF) [file pone.0111014.s001.pdf]

Brandeis University

NJ Mosquito Control Questionnaire

Please place a check mark or write in your response to all questions.

1. Does any child live in your household?

____ yes ____ no. If you have no children in your household, please leave questions asking about the selected child blank and skip to question 4.

↓

If you have only one child, consider him or her the "selected child." If you have more than one child in your household, then questions asking about the "selected child" in your household refer to your oldest child in elementary school.

2. What is the age of your selected child? ________ years (write 1 if 1 year or less)

3. What grade is your selected child in? ________ (write none if not in school)

4. How has your experience been with mosquitoes in your neighborhood this summer? Would you consider it not a problem, a mild problem, a moderate problem, a severe problem, or an extremely horrible problem? (Mark one)

a. Not a problem
b. Mild
c. Moderate
d. Severe
e. Extremely horrible

5. During a typical week this summer, how many **hours per week** did you and/or your selected child spend on your outdoor porch, steps, or yard engaged in each of the following activities? (If none, put 0)

**Activity on outdoor porch or yard**	Hours **you** spent in a typical **week**	Hours **selected child** spent in a typical **week**
a. Eating or cook out		
b. Gardening or mowing lawn		
c. Maintaining house or car		
d. Playing catch, frisbee, bocce, horseshoes, croquet, volleyball/badminton etc.		
e. Relaxing, socializing, talking, reading, hanging out, smoking		
f. Others– please specify below		

6. During a typical week this summer, to what extent did mosquitoes prevent you from enjoying outdoor recreational activities? (Mark one)

Not at all	A little bit	Somewhat	Very much

7. During a typical week this summer, to what extent did mosquitoes prevent **your selected child** from enjoying outdoor recreational activities? (Mark one)

Not at all	A little bit	Somewhat	Very much

8. We are interested in the impact of mosquitoes on outdoor porch or yard activities. During a typical week this summer, how many **hours per week would you and/or your selected child have spent** on your outdoor porch or yard if you and/or your selected child had no concerns over mosquitoes?

**Activity**	Hours **you** would have spent in a typical **week**	Hours **selected child** would have spent in a typical **week**
a. Eating or cook out		
b. Gardening or mowing lawn		
c. Maintaining house or car		
d. Playing catch, frisbee, bocce, horseshoes, croquet, volleyball/badminton etc.		
e. Relaxing, socializing, talking, reading, hanging out, smoking		
f. Others– please specify below		

9. Apart from this or any previous survey, had you ever seen or heard of a black and white striped mosquito called the Asian tiger mosquito, considered a potential carrier of diseases?
_____ Yes
_____ No

10. Has your selected child discussed the Asian tiger mosquito with you?
_____ Yes
_____ No

11. Did your selected child learn about mosquitoes in his/her elementary school?
_____ Yes
_____ No

12a. During a typical week this summer, were you and/or your selected child bitten by a mosquito while outdoors? (Mark one)

**Response**	**You**	**Selected child**
Yes, bitten once, outdoors		
Yes, bitten more than once, outdoors		
Not bitten outdoors		
Don’t know		

12b. During a typical week this summer, were you and/or your selected child bitten by a mosquito while indoors? (Mark one)

**Response**	**You**	**Selected child**
Yes, bitten once, indoors		
Yes, bitten more than once, indoors		
Not bitten indoors		
Don't know		

13. In which time period(s) were you and/or your selected child bitten by a mosquito? (Mark all that apply)

**Response**	**You**	**Selected child**
Early morning		
Late morning		
Afternoon		
Evening		
Night		
Not applicable or don't know		

14. Have you, or your selected child, done any of the following actions around your house and/or yard this summer?

**Action**	**You**			**Selected child**		
	Yes	No	NA*	Yes	No	NA*
Cleaned gutters						
Placed insecticides (such as mosquito dunks®) into standing water						
Emptied water out of outdoor containers						
Placed fish into containers outdoors						
Stored containers upside down						
Requested visits from pest control company						
Checked backyard weekly for standing water						
Drilled holes into trashcans						
Removed tires from backyard						

*NA= Not applicable (for example, you don't have a yard; the situation did not exist at the beginning of the summer; the action is not your responsibility)

15. Do you have plastic toys, pet watering dishes, and/or plant saucers outside your house?
_____ Yes
_____ No
_____ Not applicable or don't know

16. Is it possible for mosquitoes to hatch in water allowed to collect in plastic toys left outdoors?
_____ Yes
_____ No
_____ Don't know

17. In general, Standing water +______ days = Mosquitoes? (Mark one)
______ 3 days
______ 7 days
______ 14 days
______ 21 days
______ Don't know

18. Which of the following statements is true:
______ Only the male mosquito can bite
______ Only the female mosquito can bite
______ Both the male and female mosquito can bite
______ Don't know

19. **During this summer**, how much did your household pay for the following items specifically to control mosquitoes?
$_____________ Repairing leaky outdoor faucets and/or water pipes
$_____________ Repairing window and/or door screens
$_____________ Adding insecticides to standing water outside your house
$_____________ Bug zapper, mosquito trap, light magnet, mosquito magnet, propane trap
$_____________ Spray to repel mosquitoes
$_____________ Burning coils or candles to repel mosquitoes
$_____________ Other, specify_________________________

20. During a typical summer week, did you and/or your selected child use insect repellent, such as Cutter ®, during outdoor porch or yard activities as protection against mosquitoes? (Mark one)

**Response**	**You**	**Selected child**
Yes		
Sometimes		
No		

21. How many people live in your household (**including** yourself)?
____

22. How many people in your household (**including** yourself) are in the following age groups?

__________ Under 15 years
__________ 15-24
__________ 25-34
__________ 35-44
__________ 45-54
__________ 55-64
_________ 65-74
_________ 75-84
_________ over 85

23. Of the total number of children in your household, how many are currently enrolled in elementary school?
____

24. What is the gender of your selected child?
_____ Boy
_____ Girl

25. Which school does your selected child attend?
____

26. What is the level of your selected child's school?
_____ Elementary
_____ Junior high school or middle school
_____ High school
_____ Post-secondary institution (e.g. college)
_____ Others

27. In which age group are you?

__________ 15-24	_________ 55-64
__________ 25-34	_________ 65-74
__________ 35-44	_________ 75-84
__________ 45-54	_________ over 85

28. What is your gender?
_____ Male
_____ Female

29. What is your level of education? (Mark one)
_____ Less than 9th grade
_____ 9th to 12th grade (no diploma)
_____ High school graduate or equivalent
_____ Some college, no degree
_____ Associate’s degree
_____ Bachelor’s degree
_____ Graduate or professional degree

30. What is your current employment status? (Mark one)
_____ Employed full time
_____ Employed part time
_____ Active at home (homemaker, parent)
_____ Student, not working
_____ Unemployed - looking for a job
_____ Not working - not looking for a job
_____ Retired

THANK YOU FOR YOUR PARTICIPATION.
